# Cytotoxic T lymphocyte-associated protein 4 gene polymorphisms are associated with ANCA-associated vasculitis in the Guangxi population of China

**DOI:** 10.3389/fimmu.2025.1631088

**Published:** 2025-09-09

**Authors:** Kunpeng Bu, Binglan Yang, Peigeng He, Chao Xue

**Affiliations:** ^1^ Endocrinology and Metabolism Nephrology Department, Guangxi Medical University Cancer Hospital, Nanning, Guangxi, China; ^2^ Department of Nephrology, The Second Affiliated Hospital of Guangxi Medical University, Nanning, Guangxi, China

**Keywords:** CTLA-4, vasculitis, T-cell, autoimmune diseases, single-nucleotide polymorphism

## Abstract

**Introduction:**

Heredity and epigenetic factors contribute to the pathogenesis of neutrophil-cytoplasmic antibody (ANCA)-associated vasculitis (AAV). Cytotoxic T lymphocyte-associated protein 4 (CTLA-4), an inhibitory receptor regulating T-cell homeostasis and maintaining self-tolerance, has emerged as a key target for immune screening and therapeutics in autoimmunity and cancer. CTLA-4 is associated with various autoimmune diseases; however, the relationship between CTLA-4 polymorphisms and AAV in the Guangxi population of China remains underexplored. In the present case–control study, we evaluated the effects of CTLA-4 polymorphisms on AAV susceptibility in the Guangxi population of China.

**Methods:**

A total of 343 patients with AAV and 343 healthy controls were recruited. High-throughput sequencing was used to genotype *CTLA4* variants, and logistic regression analysis was used to assess their association with AAV risk. The relationship between the haplotypes of *CTLA4* single-nucleotide polymorphisms (SNPs) and AAV risk was assessed using the SHEsis platform.

**Results:**

Three *CTLA4* SNPs— *rs62182595, rs16840252*, and *rs5742909*— showed significant association with AAV susceptibility. The ATT and GCC haplotypes, comprising these loci, were also associated with an increased risk of AAV.

**Discussion:**

These findings suggest that *CTLA4* polymorphisms (*rs62182595, rs16840252*, and *rs5742909*) may contribute to AAV susceptibility in the Guangxi population and offer preliminary markers for risk assessment, early diagnosis, and personalized management of AAV.

## 1 Introduction

Anti-neutrophil cytoplasmic antibody-associated vasculitis (AAV) comprises a group of small-vessel inflammatory disorders with largely unknown pathogenesis. AAV is serologically defined by autoantibodies against two specific neutrophil antigens: proteinase 3 and myeloperoxidase (1). Pathologically, AAV is characterized by necrotizing inflammation of vessel walls. Clinically, AAV includes three principal subtypes: granulomatosis with polyangiitis (2), microscopic polyangiitis (MPA) (3, 4), and eosinophilic granulomatosis with polyangiitis (5). Although the exact pathogenesis of AAV is unknown, immune dysregulation, which is influenced by genetic and environmental factors, plays a central role in AAV pathogenesis (6). Genome-wide analyses have identified several predisposing loci, mainly within the major histocompatibility complex region. Specifically, variants in the human leukocyte antigen-DQ segment show strong genetic associations with AAV pathogenesis (7, 8). In Asia, AAV prevalence ranges from 46 to 421 per million (9), and in China, its prevalence is increasing annually, especially among the aging population. The current standard treatment for AAV involves systemic immunosuppressants targeting B and T cells, including glucocorticoids, cyclophosphamide, and rituximab (10); however, many patients with AAV remain unresponsive to the current standard of care and experience disease relapses (11). Therefore, identification of novel therapeutic targets to achieve long-term disease remission is necessary.

Cytotoxic T lymphocyte-associated protein 4 (CTLA-4), a crucial immune checkpoint molecule within the CD28 immunoglobulin superfamily, exhibits predominant expression in T lymphocytes (12). CTLA-4 is an important regulator of T-cell homeostasis and self-tolerance and can modulate the T-cell response by blocking CD28 co-stimulation of T-cells during T-cell activation (13, 14). CTLA-4 expression in T cells is tightly regulated, and the failure of the transport mechanism of CTLA-4 to the cell surface leads to autoimmune susceptibility (14). Given its central role in immune modulation, CTLA-4 has emerged as a promising therapeutic target across multiple disease domains, particularly in autoimmune conditions and oncology. Moreover, CTLA-4-targeted therapies through the successful implementation of CTLA-4 inhibitors have been validated to manage various immune-mediated disorders clinically (12). In AAV, CTLA-4–Ig fusion proteins that block T-cell co-stimulation offer a novel therapeutic strategy potentially advantageous over traditional immunosuppressive regimens (15). However, genetic studies have reported inconsistent associations between *CTLA4* polymorphisms and AAV. For instance, a European case–control study identified the association between *CTLA4* polymorphism— rs3087243 —and AAV (16). In contrast, a case—control study of the Han Chinese population in Beijing, China, reported no association between *CTLA4* polymorphisms (rs231775 and rs5742909) and AAV susceptibility (17). These findings suggest the strong influence of ethnicity, population, and regional variation on genetic susceptibility; however, the association between single-nucleotide polymorphisms (SNPs) at the *CTLA4* locus and AAV susceptibility in the Guangxi population has not been reported.

To address this gap, we conducted a case—control study involving 343 patients with AAV from Guangxi and selected three candidate *CTLA4* SNPs to evaluate their association with AAV risk in this population.

## 2 Materials and methods

### 2.1 Ethics approval

This study was approved by the Medical Ethics Committee of The Second Affiliated Hospital of Guangxi Medical University (2018 KY-0100) and was conducted in compliance with the Declaration of Helsinki. We have obtained written consent from all participants.

### 2.2 Study population characteristics

The MPA investigation cohort constituted 343 patients diagnosed with AAV retrospectively enrolled from the Second Affiliated Hospital of Guangxi Medical University (2005-2024). The cases were confirmed through rigorous diagnostic protocols aligned with the Chapel Hill Consensus Conference classification criteria (18). Exclusion criteria strictly applied to individuals presenting with: (1) secondary vasculitides induced by infectious processes, neoplastic disorders, or pharmacological agents; (2) concurrent autoimmune conditions (including rheumatoid arthritis, systemic lupus erythematosus, Henoch-Schönlein purpura). A parallel control group of 343 age- and sex-matched healthy volunteers was established, systematically excluding participants with autoimmune comorbidities or malignant histories.

### 2.3 SNP selection and DNA extraction

We selected three *CTLA4* SNPs (*rs62182595*, *rs16840252*, and *rs5742909*) based on the following criteria: 1) all SNPs had a minor allele frequency (MAF) ≥ 0.05 (19) and 2) Chinese allele or genotype data of these SNPs were available in 1000 genomes (https://grch37.ensembl.org/).

DNA extraction was performed using a Rapid Blood Genomic DNA Isolation Kit (Sangon, Shanghai, China), following the manufacturer’s instructions. The concentration of the DNA was measured using a Qubit 2.0 (Thermo Fisher Scientific, Inc.) to ensure the extraction of adequate amounts of high-quality genomic DNA.

### 2.4 Statistical analyses

All statistical analyses were conducted within a rigorous statistical framework using IBM SPSS Statistics, version 26.0 (IBM Corp., Armonk, NY, USA) (20). Initial demographic comparisons were performed using independent samples t-tests for continuous variables and chi-square (χ²) test for categorical variables. Hardy–Weinberg equilibrium (HWE) for genotype and allele distribution was assessed using both χ² and Fisher’s exact tests. Genetic association analysis was conducted using an unmatched case–control paradigm through the SNPstats analytical platform (https://www.snpstats.net/start.htm) (21), applying multivariate logistic regression with covariate adjustment (age, ethnicity, gender) to derive adjusted odds ratios (ORs) and 95% confidence intervals (CIs). Haplotype architecture reconstruction (22) was performed using the SHEsis online suite (23), incorporating permutation-based significance testing. To control the Type I in multi-group comparisons, Bonferroni multiplicity correction was applied, maintaining a family-wise significance threshold set at α=0.05.

## 3 Results

### 3.1 Demographic characteristics

The study cohort comprised equal numbers of patients with AAV (n = 343) and healthy controls (n = 343). Gender distribution showed comparable patterns across groups, with the AAV cohort comprising 130 male and 213 female participants, compared to 146 males and 197 females in the control group (P = 0.213). Age distribution revealed significant intergroup variation. The AAV group showed a mean age of 56.7 years (range, 8–86 years; interquartile range (IQR), 49–67 years), which was significantly higher than that of the control group (mean, 44.6 years, range: 18–81 years; IQR, 35–53 years) (P < 0.05). Ethnic composition was comparable between the groups, with 202 Han Chinese and 141 non-Han Chinese participants in both cohorts (P = 0.738). To ensure analytical rigor, potential confounding variables, including age, sex, and ethnicity, were incorporated as covariates in the logistic regression analysis (**Table 1**).

**Table 1:** Demographic features of AAV cases and control group.

Variable	Case (n=343)	Control (n=343)	*p*
Gender (male/female)	130/213	146/197	0.213
Ethnicity (Han/non-Han)	202/141	251/184	0.738
Age (years)	56.6(49,67) ^a^	44.6 (35,53) ^a^	<0.01*

AAV, Anti-neutrophil cytoplasmic antibody (ANCA)-associated vasculitis; n, number of people; a, The description of skewed distribution data statistics uses median (lower quartile, upper quartile) representation; *p*-value: The Student’s t-test or the Chi-square test was used to compare variables in groups; * denotes statistical significance (p<0.05).

### 3.2 Information for selected SNPs

Basic information for the selected *CTLA4* SNPs is shown in **Table 2**. All loci are located on chromosome 2, specifically within the promoter region and 5’ upstream regulatory region (URR). The MAF for each SNP was greater than 0.05, indicating that variants are common in the Guangxi population. Additionally, all SNPs conformed to HWE (p > 0.05).

**Table 2:** Basic information of the selected SNPs.

SNP	Chromosome	Position	Alleles	Gene	Location	MAF		HWE-*p*	Allele-*p*	Genotype-*p*
						Control	Case			
rs62182595	Chr2	204731188	G>A	CTLA4	promoter	0.11	0.10	0.24	0.42	0.19
rs16840252	Chr2	204731519	C>T	CTLA4	5’ URR	0.12	0.10	0.13	0.34	0.29
rs5742909	Chr2	204732347	C>T	CTLA4	promoter	0.11	0.10	0.11	0.48	0.24

AAV, Anti-neutrophil cytoplasmic antibody (ANCA)-associated vasculitis; SNP, single nucleotide polymorphism; MAF, minor allele frequency; HWE, Hardy-Weinberg equilibrium; chr, chromosome; The *p*-value was calculated using Chi-square test and Fisher’s exact test; Bonferroni correction was used for multiple comparison.

### 3.3 Correlation of SNPs with AAV susceptibility under different genetic models

The association between the SNPs and AAV risk were assessed using different genetic models (adjusted for age, sex, and race). The *rs62182595*, *rs16840252*, and *rs5742909* polymorphisms in *CTLA4* were significantly associated with AAV susceptibility in the Guangxi population under different genetic models (**Table 3**). For *rs62182595*, the GA genotype in the codominant genetic model (OR = 0.58; 95% CI [0.38–0.90]; p = 0.048), the GA-AA genotype in the dominant genetic model (0.61; 0.40–0.93; p = 0.021), and GA genotypes in the overdominant genetic model (0.58; 0.38–0.90; p = 0.014), were all associated with reduced AAV susceptibility in the Guangxi population. For *rs16840252*, the CT-TT genotype in the dominant genetic model (OR = 0.61; 95% CI [0.40–0.93]; p = 0.022) and the C/T genotype in the overdominant genetic model (0.60; 0.39–0.92; p = 0.019) were also associated with reduced risk of AAV. In contrast, only the CT genotype at *rs5742909* in the overdominant genetic model showed association with reduced susceptibility to AAV (OR =0.64; 95% CI [0.42–0.99]; p = 0.044).

**Table 3:** The relationship between the SNPs and the risk of AAV in Guangxi population in different genetic models.

SNP	Models	Genotype/Allele	Control(freq)	Case(freq)	OR (95% CI)	*p* −value
rs62182595	Allele	A	75(10.9%)	66(9.6%)	0.87(0.61-1.23)	0.42
		G	611(89.1%)	620(90.4%)		
	Codominant	G/G	270 (78.7%)	282 (82.2%)	1.00	0.048*
		G/A	71 (20.7%)	56 (16.3%)	0.58 (0.38-0.90)	
		A/A	2 (0.6%)	5 (1.5%)	1.32 (0.21-8.34)	
	Dominant	G/G	270 (78.7%)	282 (82.2%)	1.00	0.021*
		G/A-A/A	73 (21.3%)	61 (17.8%)	0.61 (0.40-0.93)	
	Recessive	G/G-G/A	341 (99.4%)	338 (98.5%)	1.00	0.68
		A/A	2 (0.6%)	5 (1.5%)	1.46 (0.23-9.17)	
	Overdominant	G/G-A/A	272 (79.3%)	287 (83.7%)	1.00	0.014*
		G/A	71 (20.7%)	56 (16.3%)	0.58 (0.38-0.90)	
rs16840252	Allele	C	606 (88.3%)	617 (89.9%)	1.18(0.84-1.66)	0.34
		T	80 (11.7%)	69 (10.1%)		
	Codominant	C/C	267 (77.8%)	280 (81.6%)	1.00	0.063
		C/T	72 (21%)	57 (16.6%)	0.60 (0.39-0.92)	
		T/T	4 (1.2%)	6 (1.8%)	0.88 (0.20-3.87)	
	Dominant	C/C	267 (77.8%)	280 (81.6%)	1.00	0.022*
		CT-TT	76 (22.2%)	63 (18.4%)	0.61 (0.40-0.93)	
	Recessive	C/C-C/T	339 (98.8%)	337 (98.2%)	1.00	0.98
		T/T	4 (1.2%)	6 (1.8%)	0.98 (0.23-4.24)	
	Overdominant	C/C-T/T	271 (79%)	286 (83.4%)	1.00	0.019*
		C/T	72 (21%)	57 (16.6%)	0.60 (0.39-0.92)	
rs5742909	Allele	C	610(88.9%)	618(90.1%)	1.13(0.80-1.60)	0.48
		T	76(11.1%)	68(9.9%)		
	Codominant	C/C	270 (78.7%)	281 (81.9%)	1.00	0.13
		C/T	70 (20.4%)	56 (16.3%)	0.64 (0.42-0.99)	
		T/T	3 (0.9%)	6 (1.8%)	1.13 (0.22-5.70)	
	Dominant	C/C	270 (78.7%)	281 (81.9%)	1.00	0.056
		C/T-T/T	73 (21.3%)	62 (18.1%)	0.66 (0.43-1.01)	
	Recessive	C/C-C/T	340 (99.1%)	337 (98.2%)	1.00	0.8
		T/T	3 (0.9%)	6 (1.8%)	1.23 (0.24-6.15)	
	Overdominant	C/C-T/T	273 (79.6%)	287 (83.7%)	1.00	0.044*
		C/T	70 (20.4%)	56 (16.3%)	0.64 (0.42-0.99)	

AAV, Anti-neutrophil cytoplasmic antibody (ANCA)-associated vasculitis; SNP, single nucleotide polymorphism; OR, odds ratio; CI, confidence interval; The p-value, OR, and 95% CI were derived from a logistic regression model adjusted for age, ethnicity, and gender; * denotes statistical significance (*p*<0.05).

### 3.4 Correlation between selected SNPs and risk of AAV development in different subgroups of the population

To assess the population subgroups in which the above *CTLA4* SNPs specifically influence the risk of developing AAV, subgroup analyses were performed by sex (male and female) and ethnicity (Han Chinese and non-Han Chinese). The analysis revealed a significant influence of sex and ethnicity on the association between the three SNPs and AAV risk. Specifically, across different genetic models, all three SNPs—*rs62182595, rs16840252*, and *rs5742909*— were significantly associated with reduced AAV susceptibility in the male subgroup (**Table 4**), whereas no significant association was observed in the female subgroup (**Supplementary Table 1**). Regarding ethnicity, the strongest protective associations were observed in the Han Chinese subgroup (**Table 5**, **Supplementary Table 2**), suggesting a population-specific genetic effect on AAV risk.

**Table 4:** The relationship between the SNPs and the risk of AAV in male of Guangxi population in different genetic models.

SNP	Models	Genotype/Allele	Control(freq)	Case(freq)	OR (95% CI)	*p* −value
rs62182595	Allele	A	35(12.0%)	20(7.7%)	0.61(0.34~1.09)	0.092
		G	257(88.0%)	240(92.3%)		
	Codominant	G/G	112 (76.7%)	111 (85.4%)	1.00	0.054
		G/A	33 (22.6%)	18 (13.8%)	0.41 (0.20-0.87)	
		A/A	1 (0.7%)	1 (0.8%)	0.37 (0.01-18.35)	
	Dominant	G/G	112 (76.7%)	111 (85.4%)	1.00	0.016*
		G/A-A/A	34 (23.3%)	19 (14.6%)	0.41 (0.20-0.86)	
	Recessive	G/G-G/A	145 (99.3%)	129 (99.2%)	1.00	0.67
		A/A	1 (0.7%)	1 (0.8%)	0.44 (0.01-20.84)	
	Overdominant	G/G-A/A	113 (77.4%)	112 (86.2%)	1.00	0.018*
		G/A	33 (22.6%)	18 (13.8%)	0.41(0.20-0.87)	
rs16840252	Allele	C	255(87.3%)	240(92.3%)	0.74(0.98~3.08)	0.055
		T	37(12.7%)	20(7.7%)		
	Codominant	C/C	110 (75.3%)	111 (85.4%)	1.00	0.034*
		C/T	35 (24%)	18 (13.8%)	0.39 (0.19-0.81)	
		T/T	1 (0.7%)	1 (0.8%)	0.36 (0.01-18.15)	
	Dominant	C/C	110 (75.3%)	111 (85.4%)	1.00	0.0093*
		C/T-T/T	36 (24.7%)	19 (14.6%)	0.39 (0.19-0.81)	
	Recessive	C/C-C/T	145 (99.3%)	129 (99.2%)	1.00	0.67
		T/T	1 (0.7%)	1 (0.8%)	0.44 (0.01-20.84)	
	Overdominant	C/C-T/T	111 (76%)	112 (86.2%)	1.00	0.011*
		C/T	35 (24%)	18 (13.8%)	0.39 (0.19-0.82)	
rs5742909	Allele	C	254(87.0%)	240(92.3%)	1.80(1.02-3.17)	0.04*
		T	38(13.0%)	20(7.7%)		
	Codominant	C/C	110 (75.3%)	111 (85.4%)	1.00	0.019*
		C/T	34 (23.3%)	18 (13.8%)	0.37 (0.18-0.78)	
		T/T	2 (1.4%)	1 (0.8%)	0.20 (0.01-5.04)	
	Dominant	C/C	110 (75.3%)	111 (85.4%)	1.00	0.0053*
		C/T-T/T	36 (24.7%)	19 (14.6%)	0.36 (0.17-0.75)	
	Recessive	C/C-C/T	144 (98.6%)	129 (99.2%)	1.00	0.37
		T/T	2 (1.4%)	1 (0.8%)	0.24 (0.01-5.90)	
	Overdominant	C/C-T/T	112 (76.7%)	112 (86.2%)	1.00	0.0087*
		C/T	34 (23.3%)	18 (13.8%)	0.38 (0.18-0.80)	

AAV, Anti-neutrophil cytoplasmic antibody (ANCA)-associated vasculitis; OR, odds ratio; CI, confidence interval; The *p*-value, OR, and 95% CI were derived from a logistic regression model adjusted for age and ethnicity; * denotes statistical significance (*p*<0.05).

**Table 5:** The relationship between the SNPs and the risk of AAV in Han of ethnicity Guangxi population in different genetic models.

SNP	Models	Genotype/Allele	Control(freq)	Case(freq)	OR (95% CI)	*p* −value
rs62182595	Allele	A	60(12.0%)	26(06.4%)	0.50(0.31~0.82)	0.0048*
		G	442(88.0%)	378(93.6%)		
	Codominant	G/G	193 (76.9%)	176 (87.1%)	1.00	0.0037*
		G/A	56 (22.3%)	26 (12.9%)	0.43 (0.25-0.75)	
		A/A	2 (0.8%)	0 (0%)	0.00 (0.00-NA)	
	Dominant	G/G	193 (76.9%)	176 (87.1%)	1.00	0.0014*
		G/A-A/A	58 (23.1%)	26 (12.9%)	0.42 (0.24-0.72)	
	Recessive	G/G-G/A	249 (99.2%)	202 (100%)	1.00	0.17
		A/A	2 (0.8%)	0 (0%)	0.00 (0.00-NA)	
	Overdominant	G/G-A/A	195 (77.7%)	176 (87.1%)	1.00	0.0026*
		G/A	56(22.3%)	26(12.9%)	0.43(0.25-0.76)	
rs16840252	Allele	C	438(87.3%)	377(93.3%)	2.04(1.27~3.27)	0.0025*
		T	64(12.7%)	27(6.7%)		
	Codominant	C/C	191 (76.1%)	175 (86.6%)	1.00	0.0029*
		C/T	56 (22.3%)	27 (13.4%)	0.45 (0.26-0.79)	
		T/T	4 (1.6%)	0 (0%)	0.00 (0.00-NA)	
	Dominant	C/C	191 (76.1%)	175 (86.6%)	1.00	0.0018*
		C/T-T/T	60 (23.9%)	27 (13.4%)	0.43 (0.25-0.74)	
	Recessive	C/C-C/T	247 (98.4%)	202 (100%)	1.00	0.064
		T/T	4 (1.6%)	0 (0%)	0.00 (0.00-NA)	
	Overdominant	C/C-T/T	195 (77.7%)	175 (86.6%)	1.00	0.0049*
		C/T	56 (22.3%)	27 (13.4%)	0.46 (0.26-0.80)	
rs5742909	Allele	C	444(88.4%)	377(93.3%)	1.82(1.13~2.94)	0.012*
		T	58(11.6%)	27(6.7%)		
	Codominant	C/C	195 (77.7%)	175 (86.6%)	1.00	0.02*
		C/T	54 (21.5%)	27 (13.4%)	0.49 (0.28-0.85)	
		T/T	2 (0.8%)	0 (0%)	0.00 (0.00-NA)	
	Dominant	C/C	195 (77.7%)	175 (86.6%)	1.00	0.0076*
		C/T-T/T	56 (22.3%)	27 (13.4%)	0.48 (0.28-0.83)	
	Recessive	C/C-C/T	249 (99.2%)	202 (100%)	1.00	0.27
		T/T	2 (0.8%)	0 (0%)	0.00 (0.00-NA)	
	Overdominant	C/C-T/T	197 (78.5%)	175 (86.6%)	1.00	0.011*
		C/T	54 (21.5%)	27 (13.4%)	0.49 (0.28-0.86)	

AAV, Anti-neutrophil cytoplasmic antibody (ANCA)-associated vasculitis; OR, odds ratio; CI, confidence interval; The *p*-value, OR, and 95% CI were derived from a logistic regression model adjusted for age and gender; * denotes statistical significance (*p*<0.05).

### 3.5 Linkage disequilibrium and haplotype analyses

**Figure 1:**
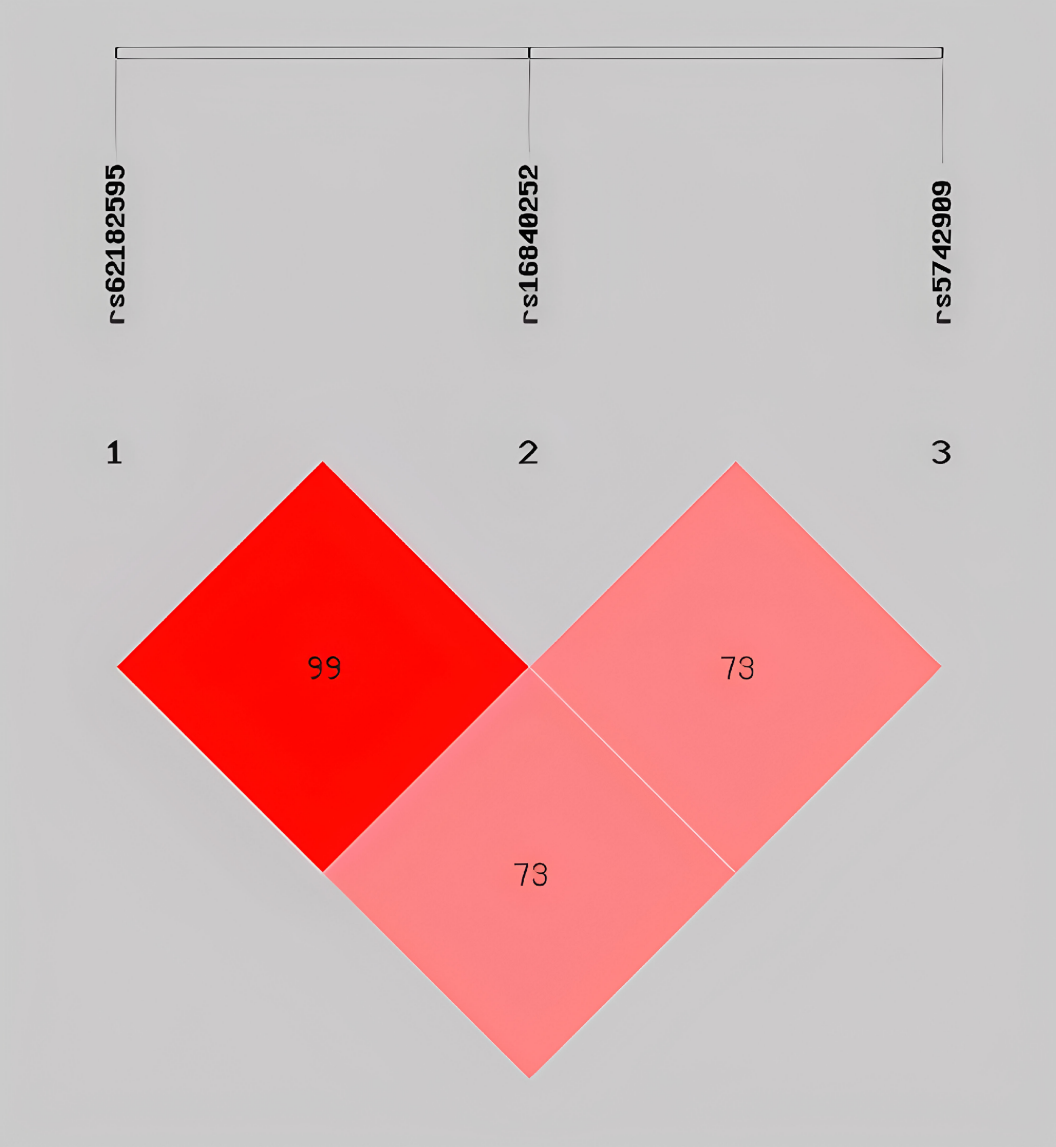
Graphical representation of the SNP locations and LD structure.

To evaluate the interactions among *CTLA4* SNPs, we performed LD and haplotype analyses using the SHEsis online platform. Strong LD was observed between *rs62182595* and *rs16840252* (D’ = 1), whereas moderate LD was observed between *rs62182595* and *rs5742909* (D’ = 0.739) and between *rs16840252* and *rs5742909* (D’ = 0.735) (**Figure 1**).

The haplotype analysis identified six haplotypes based on the three SNPs (**Table 6**). Four of these haplotypes had a frequency of less than 0.05 (24), suggesting a low frequency of occurrence in the population and unstable inheritance, and were therefore excluded from further analyses. Two haplotypes, ATT and GCC, with frequencies greater than 0.05 significantly elevated the risk of AAV development in the Guangxi population (ATT: OR=1.67, 95% CI: 1.113–2.503, p = 0.01; GCC: 1.80, 1.300–2.494, p = 0.000355).

**Table 6:** Correlations between the haplotypes of CTLA-4 gene and AAV susceptibility.

Geng	SNP	Haplotypes	Control(freq)	Case(freq)	χ 2	p	OR (95% CI)
CTLA-4	rs62182595/rs16840252/ rs5742909	ATC	33.99(0.050)	0.00(0.000)	34.958	–	–
		ATT	41.01(0.060)	66.00(0.096)	6.252	0.012437*	1.669 (1.113~2.503)
		GCC	571.01(0.832)	617.00(0.899)	12.771	0.000355*	1.801 (1.300~2.494)
		GCT	34.99(0.051)	0.00(0.000)	36.013	–	–
		GTC	5.00(0.007)	1.00(0.001)	–	–	–
		GTT	0.00(0.000)	2.00(0.003)	–	–	–

SNP, single nucleotide polymorphism; OR, odds ratio; CI, confidence interval; The p-value was calculated using Chi-square test and Fisher’s exact test; Bonferroni correction was used for multiple comparison; * denotes statistical significance (p<0.05); – denotes frequency<0.05 in both control & case has been dropped.

## 4 Discussion

The pathogenesis of AAV involves the complex interplay of host immunological dysregulation (encompassing both innate and adaptive immunity) and genetic–environmental synergism (4). The breakdown of ANCA tolerance, which is critical to disease initiation, is frequently driven by aberrant immune cell dynamics and humoral immunity deficiencies (25, 26). Nevertheless, emerging evidence from large-scale genomic investigations has substantiated the pivotal role of genetic predisposition in AAV development (7, 9, 27). In this study, we focused on identifying the risk alleles associated with AAV mechanisms and evaluated the correlation between *CTLA4* SNPs and AAV risk in the Guangxi population in China. Our findings expand the current understanding of genetic loci associated with AAV susceptibility. CTLA-4 is a master immune checkpoint receptor that delivers co-inhibitory signals crucial for attenuating excessive immune activation (28) and orchestrating immune response termination (29). Although its central regulatory role in maintaining immune homeostasis and preventing autoreactivity is well-established, the precise molecular mechanisms governing CTLA-4-mediated immune modulation remain incompletely elucidated (30). *CTLA4* polymorphisms demonstrate significant pleiotropic effects, showing associations with multiple immune-mediated pathologies including systemic lupus erythematosus, rheumatoid arthritis, autoimmune diabetes, and allograft rejection episodes (31–33).

Our data show that *CTLA4 rs62182595*, *rs16840252*, and *rs5742909* polymorphisms are significantly associated with AAV susceptibility in the Guangxi population under various genetic models. Specifically, the GA genotype of *rs62182595* in the codominant and overdominant genetic models and the GA-AA genotype in the dominant genetic model were associated with reduced AAV susceptibility in the Guangxi population. Similarly, the CT-TT and C/T genotypes of *rs16840252* (dominant genetic model) and the C/T genotype of *rs5742909* (overdominant genetic model) showed association with reduced AAV risk in the Guangxi population.

Next, we analyzed the synergistic effects of haplotype combinations to assess the possible interactions among *CTLA4* SNPs and determine the effects of these three SNPs on AAV susceptibility in the Guangxi population. Among the six haplotypes identified, ATT and GCC haplotypes significantly elevated susceptibility, suggesting an interaction between the three SNPs that increases the susceptibility to AAV in the Guangxi population, similar to the results of a previous study on CTLA-4 (34). Furthermore, previous studies suggest that SNP polymorphisms in the 5′ URR region may lead to altered selective splicing (35–37), and alterations in selective splicing can lead to variation in *CTLA4*. Therefore, we hypothesized that alterations in bases at *rs1684025* may alter the selective splicing of both the soluble CTLA-4 (sCTLA-4) and the surface full-length CTLA-4 (flCTLA-4) isoforms, ultimately affecting CTLA-4 gene expression (38). The remaining two SNPs, *rs5742909* and *rs62182595*, located in the promoter region, may lead to different levels of mRNA transcription and protein translation and affect T cell homeostasis, leading to immune dysfunction and ultimately affecting the susceptibility of individuals to AAV (39, 40). Because rs62182595*G, rs16840252*C, and rs5742909*C showed strong LD, we hypothesize that these three loci are likely to amplify the synergistic effects between the loci through gene–gene interactions (formation of different haplotypes). Such interactions could affect the expression level and protein structure of *CTLA4*, impair T-cell function, and disrupt immune homeostasis, ultimately influencing the susceptibility of individuals to AAV (41–43). Overall, our study demonstrates a significant association between *CTLA4* SNPs and AAV risk in the Guangxi population. These findings not only lay the foundation for disease risk stratification (e.g., specific genes can serve as potential genetic markers) to help guide screening of high-risk populations and provide targets for subsequent precise prevention and early intervention, but also explore the potential of *CTLA4*-targeted precision therapy. It supports the rationale for using *CTLA4* as a therapeutic target and provides evidence for “genotype-guided personalized immunotherapy.

Despite its strengths, the present study has certain limitations. First, although haplotype association analysis was conducted, we did not evaluate the expression levels of the *CTLA4* gene or protein. As gene expression is affected by numerous factors, such as transcriptional and translational regulations and gene–environmental interactions, our findings require further experimental validation. Second, sample collection was limited due to the rarity of ANCA-associated vasculitis. Third, the study population was restricted to Guangxi, China, which may limit the generalizability of the findings to other populations. In the future, it is planned to carry out cross-regional multi-center collaborative studies. While expanding the sample size, populations of different ethnic groups will be included, and the expression levels of the *CTLA4* gene and protein will be detected simultaneously. Combined with the analysis of gene-environment interactions, the research conclusions will be comprehensively verified.

## Data Availability

The raw data supporting the conclusions of this article will be made available by the authors, without undue reservation.
