# Supplementary material for: Cytotoxic T lymphocyte-associated protein 4 gene polymorphisms are associated with ANCA-associated vasculitis in the Guangxi population of China
Source: Front Immunol. 2025 Sep 9;16:1631088. doi: 10.3389/fimmu.2025.1631088 (PMC12457943; doi:10.3389/fimmu.2025.1631088)
Supplement: Supplementary file 1 [file Table1.docx]

**Supplementary Table 1: The relationship between the SNPs and the risk of AAV in female of Guangxi population in different genetic models**

| SNP | Models | Genotype/Allele | Control(freq) | Case(freq) | OR (95% CI) | *p* ‑value |
| --- | --- | --- | --- | --- | --- | --- |
| rs62182595 | Allele | A | 40(10.2%) | 46(10.8%) | 1.07(0.68-1.68) | 0.76 |
|  |  | G | 354(89.8%) | 380(89.2%) |  |  |
|  | Codominant | G/G | 158 (80.2%) | 171 (80.3%) | 1.00 | 0.40 |
|  |  | G/A | 38 (19.3%) | 38 (17.8%) | 0.73 (0.42-1.26) |  |
|  |  | A/A | 1 (0.5%) | 4 (1.9%) | 2.19 (0.21-22.34) |  |
|  | Dominant | G/G | 158 (80.2%) | 171 (80.3%) | 1.00 | 0.34 |
|  |  | G/A-A/A | 39 (19.8%) | 42 (19.7%) | 0.77 (0.45-1.32) |  |
|  | Recessive | G/G-G/A | 196 (99.5%) | 209 (98.1%) | 1.00 | 0.45 |
|  |  | A/A | 1 (0.5%) | 4 (1.9%) | 2.33 (0.23-23.61) |  |
|  | Overdominant | G/G-A/A | 159 (80.7%) | 175 (82.2%) | 1.00 | 0.24 |
|  |  | G/A | 38 (19.3%) | 38 (17.8%) | 0.72 (0.42-1.25) |  |
| rs16840252 | Allele | C | 351(89.1%) | 377(88.5%) | 0.94([0.61~1.46) | 0.79 |
|  |  | T | 43(10.9%) | 49(11.5%) |  |  |
|  | Codominant | C/C | 157 (79.7%) | 169 (79.3%) | 1.00 | 0.68 |
|  |  | C/T | 37 (18.8%) | 39 (18.3%) | 0.79 (0.46-1.36) |  |
|  |  | T/T | 3 (1.5%) | 5 (2.4%) | 1.09 (0.22-5.36) |  |
|  | Dominant | C/C | 157 (79.7%) | 169 (79.3%) | 1.00 | 0.43 |
|  |  | C/T-T/T | 40 (20.3%) | 44 (20.7%) | 0.81 (0.48-1.37) |  |
|  | Recessive | C/C-C/T | 194 (98.5%) | 208 (97.7%) | 1.00 | 0.87 |
|  |  | T/T | 3 (1.5%) | 5 (2.4%) | 1.14 (0.23-5.58) |  |
|  | Overdominant | C/C-T/T | 160 (81.2%) | 174 (81.7%) | 1.00 | 0.38 |
|  |  | C/T | 37 (18.8%) | 39 (18.3%) | 0.79 (0.46-1.35) |  |
| rs5742909 | Allele | C | 356(90.4%) | 378(88.7%) | 0.84(0.54~1.32) | 0.44 |
|  |  | T | 38(9.6%) | 48(11.3%) |  |  |
|  | Codominant | C/C | 160 (81.2%) | 170 (79.8%) | 1.00 | 0.55 |
|  |  | C/T | 36 (18.3%) | 38 (17.8%) | 0.88 (0.51-1.52) |  |
|  |  | T/T | 1 (0.5%) | 5 (2.4%) | 2.86 (0.29-28.43) |  |
|  | Dominant | C/C | 160 (81.2%) | 170 (79.8%) | 1.00 | 0.82 |
|  |  | C/T-T/T | 37 (18.8%) | 43 (20.2%) | 0.94 (0.55-1.60) |  |
|  | Recessive | C/C-C/T | 196 (99.5%) | 208 (97.7%) | 1.00 | 0.32 |
|  |  | T/T | 1 (0.5%) | 5 (2.4%) | 2.92 (0.29-29.02) |  |
|  | Overdominant | C/C-T/T | 161 (81.7%) | 175 (82.2%) | 1.00 | 0.61 |
|  |  | C/T | 36 (18.3%) | 38 (17.8%) | 0.87 (0.50-1.50) |  |

NOTE: AAV: Anti-neutrophil cytoplasmic antibody (ANCA)-associated vasculitis; OR: odds ratio; CI: confidence interval; The *p*-value, OR, and 95% CI were derived from a logistic regression model adjusted for age and ethnicity.

**Supplementary Table 2: The relationship between the SNPs and the risk of AAV in Non-Han of ethnicity Guangxi population in different genetic models**

| SNP | Models | Genotype/Allele | Control(freq) | Case(freq) | OR (95% CI) | *p* ‑value |
| --- | --- | --- | --- | --- | --- | --- |
| rs62182595 | Allele | A | 15(8.2%) | 40(14.2%) | 1.87(1.00~3.48) | 0.049* |
|  |  | G | 169(91.8%) | 242(85.8%) |  |  |
|  | Codominant | G/G | 77 (83.7%) | 106 (75.2%) | 1.00 | 0.19 |
|  |  | G/A | 15 (16.3%) | 30 (21.3%) | 1.07 (0.51-2.26) |  |
|  |  | A/A | 0 (0%) | 5 (3.5%) | NA (0-NA) |  |
|  | Dominant | G/G | 77 (83.7%) | 106 (75.2%) | 1.00 | 0.58 |
|  |  | G/A-A/A | 15 (16.3%) | 35 (24.8%) | 1.23 (0.59-2.54) |  |
|  | Recessive | G/G-G/A | 92 (100%) | 136 (96.5%) | 1.00 | 0.071 |
|  |  | A/A | 0 (0%) | 5 (3.5%) | NA (0.00-NA) |  |
|  | Overdominant | G/G-A/A | 77 (83.7%) | 111 (78.7%) | 1.00 | 0.93 |
|  |  | G/A | 15 (16.3%) | 30 (21.3%) | 1.03 (0.49-2.17) |  |
| rs16840252 | Allele | C | 168(91.3%) | 240(85.1%) | 0.54(0.30~1.00) | 0.047* |
|  |  | T | 16(8.7%) | 42(14.9%) |  |  |
|  | Codominant | C/C | 76 (82.6%) | 105 (74.5%) | 1.00 | 0.16 |
|  |  | C/T | 16 (17.4%) | 30 (21.3%) | 1.03 (0.49-2.13) |  |
|  |  | T/T | 0 (0%) | 6 (4.3%) | NA (0.00-NA) |  |
|  | Dominant | C/C | 76 (82.6%) | 105 (74.5%) | 1.00 | 0.63 |
|  |  | C/T-T/T | 16 (17.4%) | 36 (25.5%) | 1.19 (0.58-2.43) |  |
|  | Recessive | C/C-C/T | 92 (100%) | 135 (95.7%) | 1.00 | 0.055 |
|  |  | T/T | 0 (0%) | 6 (4.3%) | NA (0.00-NA) |  |
|  | Overdominant | C/C-T/T | 76 (82.6%) | 111 (78.7%) | 1.00 | 0.96 |
|  |  | C/T | 16 (17.4%) | 30 (21.3%) | 0.98 (0.47-2.03) |  |
| rs5742909 | Allele | A | 166(90.2%) | 241(85.5%) | 0.63(0.35~1.15) | 0.13 |
|  |  | G | 18(9.8%) | 41(14.5%) |  |  |
|  | Codominant | C/C | 75 (81.5%) | 106 (75.2%) | 1.00 | 0.65 |
|  |  | C/T | 16 (17.4%) | 29 (20.6%) | 1.07 (0.51-2.24) |  |
|  |  | T/T | 1 (1.1%) | 6 (4.3%) | 2.63 (0.28-24.40) |  |
|  | Dominant | C/C | 75 (81.5%) | 106 (75.2%) | 1.00 | 0.66 |
|  |  | C/T-T/T | 17 (18.5%) | 35 (24.8%) | 1.17 (0.58-2.38) |  |
|  | Recessive | C/C-C/T | 91 (98.9%) | 135 (95.7%) | 1.00 | 0.36 |
|  |  | T/T | 1 (1.1%) | 6 (4.3%) | 2.59 (0.28-23.97) |  |
|  | Overdominant | C/C-T/T | 76 (82.6%) | 112 (79.4%) | 1.00 | 0.91 |
|  |  | C/T | 16 (17.4%) | 29 (20.6%) | 1.04 (0.50-2.18) |  |

NOTE: AAV: Anti-neutrophil cytoplasmic antibody (ANCA)-associated vasculitis; OR: odds ratio; CI: confidence interval; The *p*-value, OR, and 95% CI were derived from a logistic regression model adjusted for age and ethnicity; * denotes statistical significance (*p*<0.05).
